# Association of haptoglobin phenotype with incident acute myocardial infarction in Chinese patients with type 2 diabetes

**DOI:** 10.1186/s12933-019-0867-4

**Published:** 2019-05-30

**Authors:** Resham L. Gurung, M. Yiamunaa, Sylvia Liu, Jian Jun Liu, Clara Chan, Robin Wai Munn Choo, Keven Ang, Chee Fang Sum, Subramaniam Tavintharan, Su Chi Lim

**Affiliations:** 1 Clinical Research Unit, Khoo Teck Puat Hospital, Singapore, Singapore; 2 Geriatric Education and Research Institute, Singapore, Singapore; 3 Diabetes Centre, Admiralty Medical Centre, Singapore, Singapore; 4 Saw Swee Hock School of Public Health, National University of Singapore, Singapore, Singapore

**Keywords:** Haptoglobin polymorphism, Type 2 diabetes, Acute myocardial infarction

## Abstract

**Background:**

Haptoglobin (Hp) is an abundant plasma protein with anti-oxidant properties. Hp polymorphism is associated with cardio-metabolic dysfunction but the allele conferring risk of developing acute myocardial infarction (AMI) in type 2 diabetes (T2D) patients is unclear. This study aimed to investigate the association of Hp phenotype (Hp 1-1, 2-1 and 2-2) with incident AMI in Chinese T2D patients.

**Methods:**

This prospective study included Chinese T2D participants from the Singapore Study of Macro-angiopathy and Micro-vascular Reactivity in Type 2 Diabetes (SMART2D) and Diabetic Nephropathy (DN) cohorts. Information on incidence of non-fatal AMI was collected by data linkage with the Singapore Myocardial Infarction Registry. Hp phenotype was determined using enzyme-linked immunosorbent assay. Cox proportional hazards regression models were used to evaluate the association of Hp phenotype with incident AMI, adjusted for traditional risk factors separately in two cohorts, then meta-analysed.

**Results:**

In total, 2324 Chinese participants (SMART2D; N = 1034, mean age [SD] of 59 [11]) and (DN: N = 1290, mean age [SD] of 58 [12]) were included in this study. There were total of 30 (56 events per 10,000 patient-years) and 99 (128 events per 10,000 patient-years) AMI events in SMART2D and DN cohorts respectively. In meta-analysis, presence of Hp 1 allele conferred 43% (hazard ratio [HR] = 1.43 [95% CI 1.10–1.87], P = 0.008, P_het_ = 0.413) increased risk of incident AMI, independent of age, sex, smoking, body mass index, HbA1c, diabetes duration, lipids, hypertension, renal function and usage of insulin and RAS antagonist. In adjusted model, compared to Hp 2-2 groups, individuals with Hp 1-1 (HR = 2.18 [95% CI 1.19–3.76], P = 0.010, P_het_ = 0.193) and Hp 2-1 (HR = 1.45 [95% CI 0.98–2.14], P = 0.065, P_het_ = 0.576) were at a higher risk of incident AMI. Moreover, compared to Hp 2-2 groups, non-Hp 2-2 groups (Hp 1-1 and Hp 2-1) were at 55% increased risk of incident AMI (HR = 1.55 [95% CI 1.07–2.24], P = 0.021, P_het_ = 0.940).

**Conclusions:**

Hp 1-1 phenotype was associated with increased risk of incident AMI, independent of traditional risk factors, in Chinese patients with T2D. Hp phenotyping may allow for identification of T2D individuals at higher risk for onset of AMI. However, further studies are needed to understand the underlying mechanism between Hp alleles and risk for AMI.

**Electronic supplementary material:**

The online version of this article (10.1186/s12933-019-0867-4) contains supplementary material, which is available to authorized users.

## Background

Cardiovascular disease (CVD) is a major complication of type 2 diabetes (T2D) and the leading cause of death [1, 2]. The incidence of acute myocardial infarction (AMI) is higher in T2D and [3] survival after incident event is remarkably low [4, 5]. Dyslipidemia, hypertension, smoking and diabetes are well known risk factors for AMI [6, 7]. However, individuals without any major risk factors also sometimes experience AMI [8, 9]. T2D patients are extremely heterogeneous with diverse susceptibility to cardiovascular disease. Given the growing prevalence of T2D in Asians, including Singapore [10], illuminating factors associated with increased risk of developing AMI remains essential.

Haptoglobin (Hp), an acute-phase α-glycoprotein, binds to free circulating haemoglobin, mediating its removal, thus preventing oxidative tissue damage [11]. The HP gene codes for two common alleles Hp 1 and Hp 2, yielding three genotypes or phenotypes (Hp 1-1, Hp 2-1 and Hp 2-2). These Hp polymers differ in biological properties and function [11]. Many studies have explored the role of Hp phenotype with CVD risk but the results have been conflicting. In T2D patients of non-Asian ancestry, there have been conflicting reports of both association [12, 13] and non-association [14] of Hp 2 allele with increased risk for CVD, while in type 1 diabetes patients, Costacou et al. showed that Hp 1 allele is a risk factor for CVD [15]. Importantly, Wang et al. recently found that Hp 1 allele conferred risk for macroangiopathy in Chinese patients with T2D [16].

To our knowledge, assessment of association of Hp phenotype specifically with development of AMI in a large T2D population has yet to be conducted. We, thus, aimed to prospectively evaluate the presence of an association between Hp phenotype and incidence of AMI in Chinese patients with T2D in Singapore.

## Methods

### Study population

**Fig. 1:**
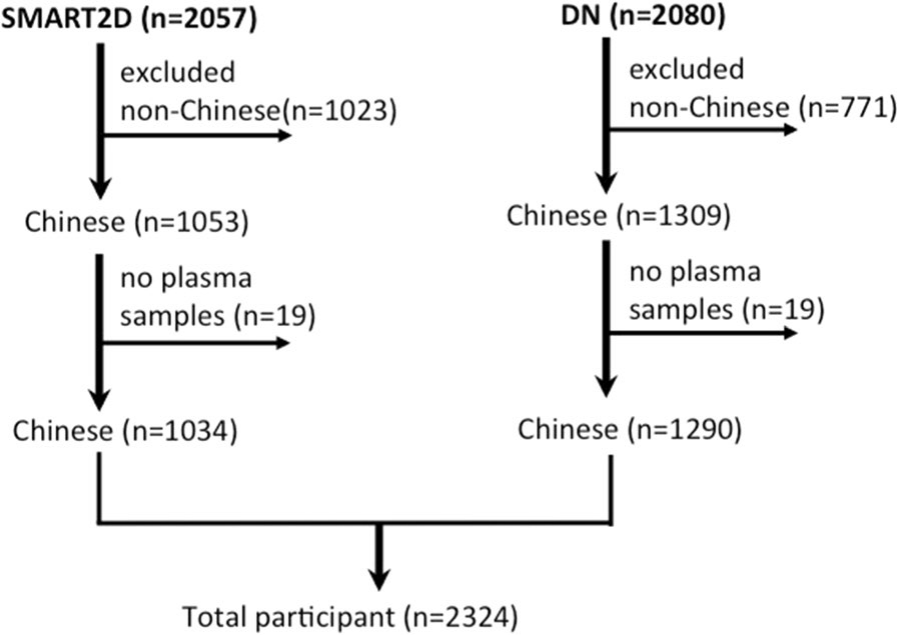
Flow diagram of study population

This study included Chinese type 2 diabetes participants in the Singapore Study of Macro-angiopathy and Micro-vascular Reactivity in Type 2 Diabetes (SMART2D) and Diabetes Nephropathy (DN) cohorts [17, 18]. In brief, SMART2D is a prospective cohort with the objective to study risk factors for vascular complications in patients with T2D. 2057 outpatients with T2D (Chinese N = 1053, Malay N = 483, Asian Indians N = 443, others N = 78) were recruited consecutively from a secondary hospital and a primary care medical facility in Singapore between August 2011 and March 2014. DN cohort recruited 2080 outpatient participants with T2D from the diabetes centre (Chinese N = 1309, Malay = 411, Asian Indian = 360) in a regional hospital between March 2004 and December 2015 with the primary aim to study the development and progression of DN and the secondary aim to study the major cardiovascular diseases (CVD) in T2D patients. For both cohorts, potential candidates were identified mainly by usage of hypoglycaemic medications in medical records or by referral from their attending physicians. T2D was diagnosed by attending physician according to prevailing American Diabetes Association criteria after exclusion of type 1 diabetes mellitus (T1DM) and diabetes due to specific causes. Diabetic patients with pregnancy, manifest infection, autoimmune disease and cancer on active treatments, and those with end stage renal disease (baseline eGFR < 15 mL/min/1.73 m^2^) were excluded. Given the vast differences in distribution of Hp alleles and the risk of CVD across ethnic groups [11, 17, 19], we focused on the association of Hp phenotypes with incident AMI in Chinese only in the current work. Participant selection for the current analysis has been illustrated in Fig. 1. The final participant number consisted of 2324 T2D patients. Written consent was obtained from each participant. The study has been approved by the Singapore National Healthcare Group Domain Specific Review Board.

### Data linkage and clinical outcome assessment

The clinical endpoint was incident acute myocardial infarction (AMI). The event was obtained via data linkage with Singapore Myocardial Infarction Registry (SMIR) [20]. The epidemiological data in SMIR included all MI diagnosed by a certified doctor with at least 2 of the following three conditions; (1) symptoms of AMI; (2) raised cardiac enzymes; (3) abnormal electrocardiogram (ECG) in all public hospitals and private hospitals [21].

### Measurement of clinical variables

Baseline clinical data and biochemical measurements for SMART2D [18] and DN cohorts [17] have been described previously. Briefly, HbA1c was quantified by the immunoturbidimetric method (Cobas Integra 800 Analyzer; Roche, Basel, Switzerland) in the DN study and measured by a point-of-care immunoassay analyzer (DCA Vantage Analyzer; Siemens, Munchen, Germany) in the SMART2D study. Triacylglycerol, high-density lipoprotein cholesterol, and low-density lipoprotein cholesterol levels were measured by enzymatic methods. Urine albumin was quantified by a solid-phase competitive chemiluminescent immunoassay (Immulite; DPC, Gwynedd, UK), and albuminuria level was presented as albumin-to-creatinine ratio (ACR, mg/g). All eGFR readings in the current studies were estimated by the Chronic Kidney Disease-Epidemiology Collaboration formula. Smoking status and diabetes duration were self-reported. Information on medication usage was extracted from the electronic medical records.

### Haptoglobin phenotyping

Plasma (10 µL) aliquots from bio-banked blood samples from the participants in this study, were subjected to Hp phenotyping using enzyme-linked immunosorbent assay (ELISA) accordingly to manufacturer’s instruction [22]. Compared to haptoglobin phenotyping using protein gel electrophoresis, ELISA based phenotyping procedure used in the current study was reported to have demonstrated sensitivity of 99.0%, 97.4% and 92.8%, and specificity of 98.1%, 97.7% and 99.8% for Hp 2-2, Hp 2-1 and Hp 1-1, respectively [22]. Separately, in a subset of Chinese participants from the DN cohort (n = 221), haptoglobin genotype was determined previously [23] using PCR method [24]. Compared to PCR method, the ELISA procedure demonstrated sensitivity of 95.1%, 98.9% and 100% and specificity of 99.2%, 98.5% and 98.4% for Hp 2-2, Hp 2-1 and Hp 1-1, respectively. Collectively, these data demonstrate ELISA procedure equivalency accuracy to gold standard methods of Hp phenotyping. Same individual with no knowledge of the participants' information performed all Hp phenotyping.

### Statistical analysis

Baseline continuous variables with normal distribution were expressed as the mean ±  standard deviation (SD), while non-normally distributed variables were presented as medians (interquartile range). Categorical data were expressed as proportions. Comparison among Hp groups was performed using ANOVA for normally distributed variables or Kruskal–Wallis test for non-normally distributed variables. The differences between study cohorts were compared by Independent sample t-test for normally distributed variables or the Mann–Whitney U test for non-normally distributed variables. Differences in proportion among the Hp groups were compared using chi‐squared tests where appropriate.

Our primary analysis of interest was a meta-analysis across the 2 study groups; SMART2D and DN. Non-normally distributed clinical variables were natural log-transformed for analysis. Cox proportional hazards regression models were used to evaluate the association of Hp phenotype with non-fatal AMI after adjustment for covariates (Model 1: adjusted for age, sex and smoking status, Model 2: further adjustment for diabetes duration, HbA1c level, body mass index, systolic blood pressure, HDL and LDL cholesterol, Model 3: further adjustment for baseline eGFR and uACR, Model 4: further adjustment for insulin and RAS antagonist usage).

We utilized an additive genetic model, which assumed a similar increase (or decrease) in the hazard ratio (HR) for each copy of the coded allele (Hp 1). Under the additive model, for the HR of 1.20 and a 2-sided significance level of P < 0.05, the estimated power of SMART2D (n = 1034) and DN cohort (n = 1290) was approximately 60%. To achieve adequate power (> 80%) for the current analysis, we first performed analysis in individual cohort, followed by meta-analysis (n = 2324) using inverse-variance-weighted, fixed-effects meta-analysis (all P for heterogeneity > 0.10). Additionally, we also compared the incident AMI risk associated with Hp 1-1 and Hp 2-1, using Hp 2-2 phenotype as a reference. Proportionally assumptions were tested based on Schoenfeld residuals. Statistical analysis was performed using Stata version 14 (StataCorp LP, College Station, TX, USA) and SPSS Version 22 (IBM Corp., Armonk, NY). A two-sided P value < 0.05 was considered statistically significant.

## Results

### Baseline profile of participants

The clinical characteristics of the participants in this study from SMART2D (mean [SD] age, 59 [11]) and DN (mean [SD] age, 58 [12]) cohorts by Hp phenotype are shown in Table 1. The phenotype prevalence were similar in two cohorts (P = 0.966); 11% for Hp 1-1, 43% for Hp 2-1, 46% for Hp 2-2 in SMART2D and 10%, 44% and 46% respectively in DN cohort (Additional file 1: Table S1). In both study cohorts, the Hp common alleles were in Hardy–Weinberg equilibrium (P > 0.05).

**Table 1:** Baseline participant characteristic by Hp phenotypes

Variable	SMART2D				DN			
	Hp 1-1 (N = 110)	Hp 2-1 (N = 449)	Hp 2-2 (N = 475)	P value	Hp 1-1 (N = 133)	Hp 2-1 (N = 561)	Hp 2-2 (N = 596)	P value
Age (years)	58.13 ± 10.16	59.05 ± 11.64	58.73 ± 11.51	0.737	58.54 ± 12.94	57.70 ± 12.64	59.10 ± 12.22	0.163
Female (%)	40.9	47.2	45.5	0.486	41.4	34.8	39.6	0.154
Smoking history (%)	–	–	–	0.788	–	–	–	0.932
Current	8.2	7.8	9.1	–	14.0	13.8	11.5	–
Ex	9.1	7.6	7.8	–	15.5	16.4	15.8	–
Never	82.7	84.6	83.1	–	70.5	69.9	72.7	–
BMI (kg/m^2^)	26.15 ± 4.12	26.75 ± 5.00	26.27 ± 4.47	0.231	26.18 ± 4.68	26.07 ± 5.26	26.21 ± 4.41	0.895
HbA1c (%)	*8.14 ± 1.69*	*7.61 ± 1.43*	*7.81 ± 1.60*	*0.018*	8.06 ± 1.82	8.31 ± 1.96	8.13 ± 1.81	0.178
Diabetes duration (years)	11.12 ± 7.63	12.15 ± 9.88	12.60 ± 10.06	0.346	12.24 ± 10.00	11.83 ± 8.90	11.92 ± 9.05	0.894
SBP (mm Hg)	140.35 ± 17.40	143.05 ± 18.61	141.62 ± 20.47	0.323	135.81 ± 19.60	134.46 ± 18.78	137.16 ± 20.20	0.067
DBP (mm Hg)	77.64 ± 8.35	78.99 ± 9.45	78.53 ± 9.67	0.383	77.53 ± 11.51	76.53 ± 10.29	76.76 ± 10.98	0.634
TC (mmol/L)	4.42 ± 1.05	4.40 ± 0.89	4.41 ± 0.95	0.954	4.60 ± 0.96	4.63 ± 1.16	4.63 ± 1.11	0.947
HDL (mmol/L)	1.36 ± 0.36	1.32 ± 0.36	1.34 ± 0.36	0.419	1.33 ± 0.40	1.28 ± 0.36	1.29 ± 0.38	0.375
LDL (mmol/L)	2.73 ± 0.89	2.70 ± 0.79	2.72 ± 0.81	0.942	2.78 ± 0.84	2.73 ± 0.86	2.74 ± 0.79	0.775
TG (mmol/L)	1.65 ± 0.98	1.73 ± 1.32	1.68 ± 1.75	0.829	1.70 ± 1.05	1.93 ± 1.44	1.80 ± 1.21	0.116
uACR (mg/g)	24.0 (7.0–103.0)	21.0 (5.8–132.3)	25.0 (7.0–116.5)	0.847	39.4 (12.0–109.5)	44.0 (11.0–239.0)	37.0 (10.0–228)	0.322
eGFR (ml/min/1.73 m^2^)	84.59 ± 28.07	83.16 ± 28.61	84.82 ± 26.92	0.649	75.52 ± 27.86	78.69 ± 29.90	77.30 ± 29.01	0.467
Medication (%)								
Insulin	22.7	27.4	24.8	0.505	26.3	34.8	30.4	0.097
RAS antagonist	55.5	62.1	58.9	0.364	60.2	64.5	46.6	0.589
Lipid lowering	80.0	82.0	83.8	0.573	71.8	73.7	74.9	0.731

Statistically significant are in italics (P values < 0.05)

Data are presented as frequencies (%) for categorical variables and as the mean ± SD for continuous, normally distributed variables

*BMI* body mass index, *DBP* diastolic blood pressure, *HDL-C* high-density lipoprotein cholesterol, *LDL-C* low-density lipoprotein cholesterol, *RAS* renin–angiotensin system, *SBP* systolic blood pressure, *TC* total cholesterol, *TG* triglycerides, *uACR* urine albumin-to-creatinine ratio, *eGFR* estimated glomerular filtration rate

P-values are for the difference among the Hp phenotype within the study cohorts

There were no significant differences in clinical profile among Hp groups within the cohorts except for HbA1c level in SMART2D (Table 1). Compared to SMART2D cohorts, the participants in DN cohorts were older, had higher proportion of male sex and smokers, more use of insulin and RAS antagonist, higher HbA1c level, higher total cholesterol, triglyceride levels, lower HDL level and poorer renal functions (higher uACR and lower eGFR) (Additional file 1: Table S1).

### Association of Hp phenotype with acute myocardial infarction

Among the study participants, 30 participants in SMART2D cohort had incident AMI in 5.7 (IQR = 4.9–6.1) years follow-up (crude incidence of 56 events per 10,000 patient-years) and 99 participants in DN cohort had incident AMI in 6.8 (IQR = 4.4–8.3) years follow-up (crude incidence of 128 events per 10,000 patient-years).

**Fig. 2:**
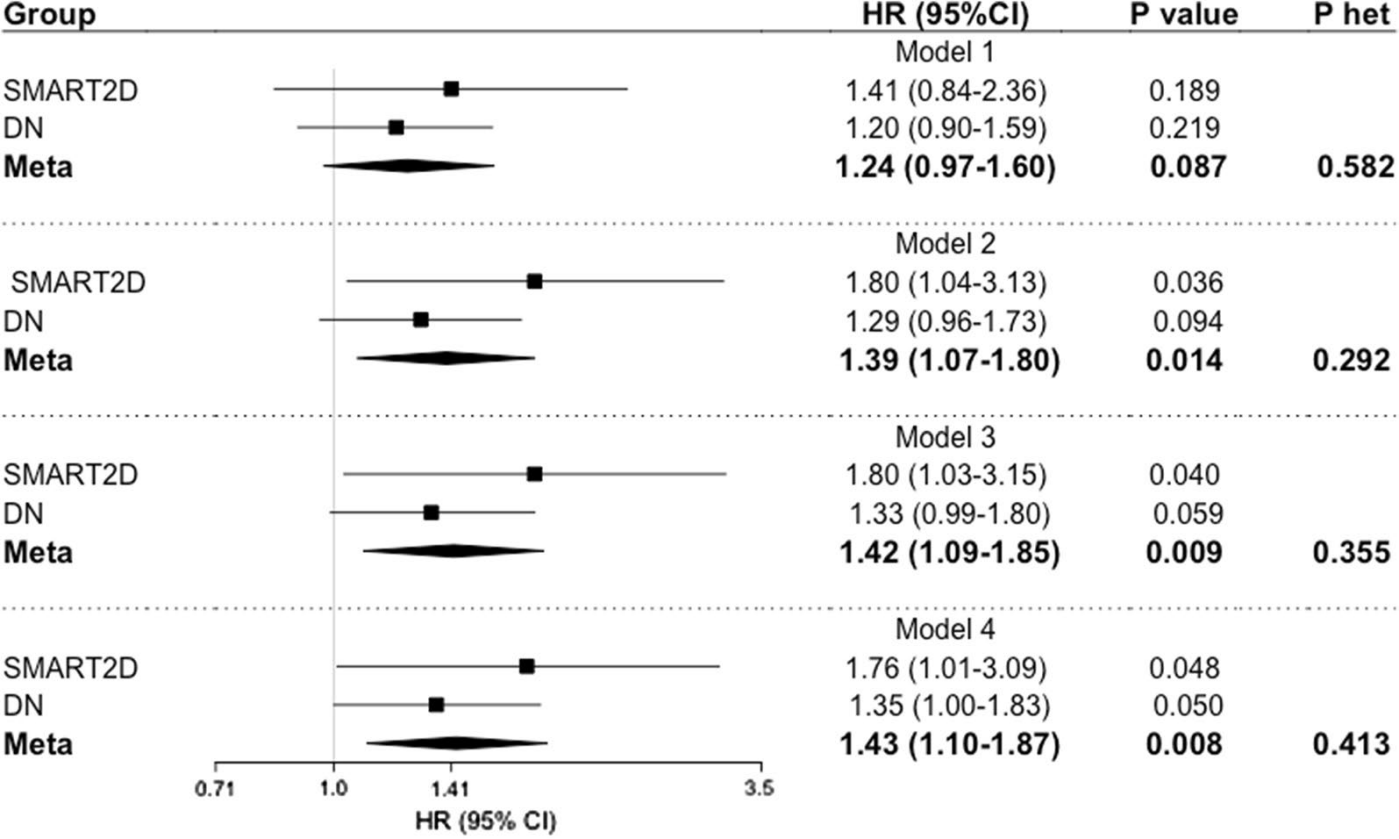
Association of Hp 1 allele with incident AMI under additive model. Data represents hazard ratio (HR) and 95% CI adjusted for traditional risk factors from SMART2D and DN cohort. Model 1: adjusted for age, sex and smoking status. Model 2: Model 1 and further adjustment for diabetes duration, HbA1c, body mass index, SBP, HDL and LDL. Model 3: Model 2 and further adjustment for eGFR and uACR. Model 4: Model 3 and further adjustment for insulin and RAS antagonist usage. Phet, Cochran’s Q heterogeneity P-value. Bold values represent statistically significant data

Figure 2 represents the results of multiple Cox proportional hazard regression analysis for both cohort and meta-analysis, in an additive model (taking Hp 1 allele as risk factor). In the meta-analysis, the presence of Hp 1 allele was associated with higher risk of AMI (HR = 1.24 [95% CI 0.97–1.60], P = 0.087, P_het_ = 0.582; adjusted for age, sex, smoking status). Further adjustment for HbA1c level, diabetes duration, systolic blood pressure, lipids, body mass index, renal functions and medication intake did not materially change the outcome (HR = 1.43 [95% CI 1.10–1.87], P = 0.008, P_het_ = 0.413; Model 4). Separately, in fully adjusted model, Hp 1 allele conferred risk of incident AMI in both SMART2D (HR = 1.76 [95% CI 1.01–3.09], P = 0.048) and DN cohort (HR = 1.35 [95% CI 1.00–1.83], P = 0.050).

**Fig. 3:**
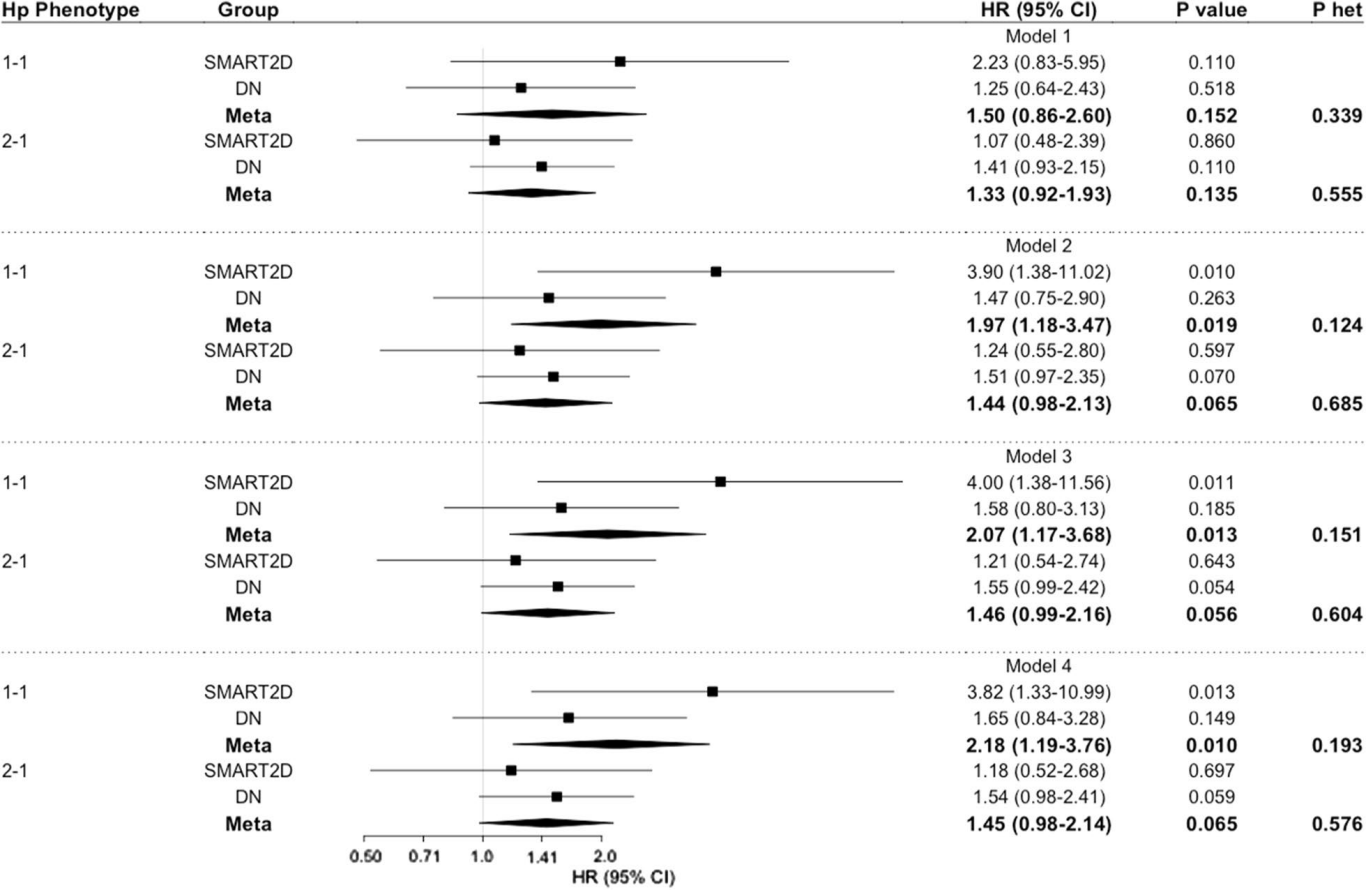
Association of Hp phenotype with incident AMI. Data represents hazard ratio (HR) and 95% CI adjusted for traditional risk factors from SMART2D and DN cohort with Hp 2-2 phenotype as reference. Model 1: adjusted for age, sex and smoking status. Model 2: Model 1 and further adjustment for diabetes duration, HbA1c, body mass index, SBP, HDL and LDL. Model 3: Model 2 and further adjustment for eGFR and uACR. Model 4: Model 3 and further adjustment for insulin and RAS antagonist usage. *Hp* haptoglobin, *P het* Cochran’s Q heterogeneity P-value; Bold values represent statistically significant data

**Fig. 4:**
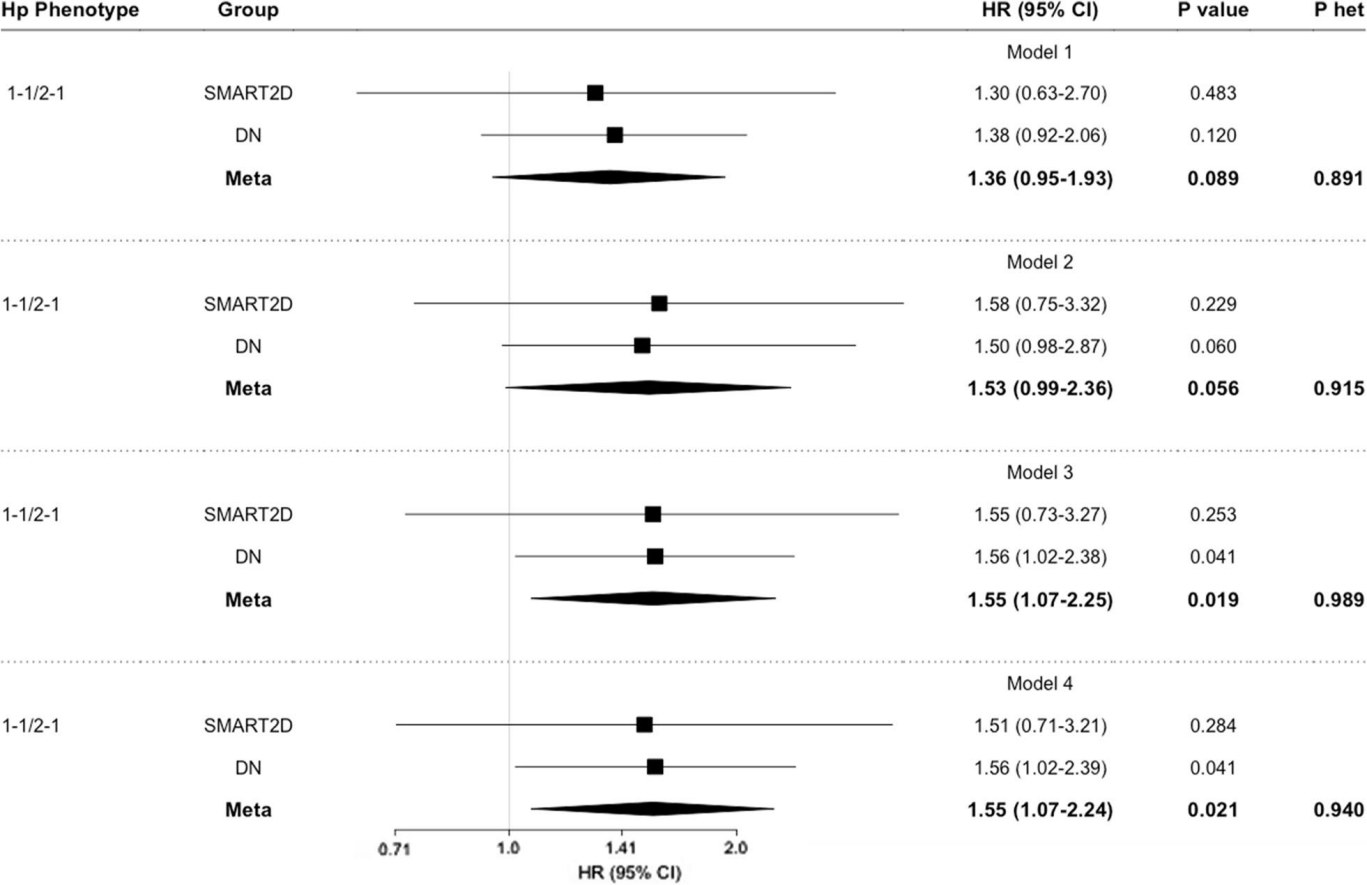
Association of Hp phenotype with incident AMI. Data represents hazard ratio (HR) and 95% CI adjusted for traditional risk factors from SMART2D and DN cohort comparing non-Hp 2-2 (Hp 1-1 and Hp 2-1) phenotype with Hp 2-2 phenotype as reference. Model 1: adjusted for age, sex and smoking status. Model 2: Model 1 and further adjustment for diabetes duration, HbA1c, body mass index, SBP, HDL and LDL. Model 3: Model 2 and further adjustment for eGFR and uACR. Model 4: Model 3 and further adjustment for insulin and RAS antagonist usage. *Hp* haptoglobin, *P het* Cochran’s Q heterogeneity P-value. Bold values represent statistically significant data

Next, we evaluated the risk associated with Hp 1-1 or Hp 2-1 compared to Hp 2-2 phenotype. From the meta-analysis, individuals with Hp 1-1 phenotype (HR = 2.18 [95% CI 1.19–3.76], P = 0.010; P_het_ = 0.193) and Hp 2-1 phenotype (HR = 1.45 [95% CI 0.98–2.14], P = 0.065, P_het_ = 0.576) were at higher risk of AMI after adjusting for traditional risk factors (Fig. 3). Additionally, we compared non Hp 2-2 group (Hp 1-1 and Hp 2-1) vs. Hp 2-2 group and observed that in a fully adjusted model, presence of Hp 1 allele is associated with 55% increased risk of incident AMI (HR = 1.55 [95% CI 1.07–2.24], P = 0.021, P_het_ = 0.940; Model 4) (Fig. 4).

## Discussion

In this prospective study, Chinese type 2 diabetes patients with Hp 1 allele were at increased risk of incident AMI, independent of traditional risk factors; including age, sex, smoking, duration of diabetes, systolic blood pressure, lipids, baseline renal function and usage of insulin and RAS antagonist. Individuals with Hp 1-1 phenotype were at double the risk of incident AMI as compared to Hp 2-2 phenotype. However, the mechanisms underlying the elevated AMI risk associated with Hp phenotype remains to be fully elucidated.

Diabetes is a risk factor for AMI with diabetic individuals having up to five-fold increased risk for incident AMI compared to non-diabetic individual [25, 26]. Diabetic patients are exposed to greater CVD risk factors including hypertension, hyperlipidemia, endothelial dysfunction and oxidative stress, which accelerate atherosclerosis [27–30]. Haptoglobin phenotypes differ in structure and biological effects, with Hp 2-2 phenotype reported to have reduced anti-oxidant properties [31, 32] that is further accentuated when hemoglobin is glycosylated [33, 34]. Several [12, 35–38] but not all [14] studies have shown that diabetes interacts with Hp phenotype and association of Hp phenotype (Hp 2-2) with CVD is observed only in diabetic population. Interestingly, recent report by Bao et al. demonstrated that plasma haptoglobin predicted incident diabetes mellitus (DM) but not CVD in general population [39]. However, this is in contrast to previous report from AMORIS study where it was shown that in general population, individual with elevated serum haptoglobin level have about four fold increased risk of AMI [40]. However, in both studies Hp phenotyping was not performed to determine the association of Hp phenotype with DM and CVD.

Elevated LDL cholesterol is a risk factor for AMI [27] and several lines of evidence have shown that Hp 2 allele and single nucleotide polymorphism (SNP) rs2000999, located within 15 kb of *HP* gene are associated with elevated levels of total cholesterol and LDL cholesterol in European [14, 41–44] and in East Asians, including Singapore Chinese population [19, 45], and are suggested to act via different pathways in modulating total cholesterol and LDL cholesterol level [42, 45]. Boettger et al. proposed that higher LDL levels in Hp 2-2 phenotype could be attributed to less efficient binding capacity of Hp 2-2 haptoglobin form to APOE, impairing the clearance of LDL cholesterol [32, 42]. However, in current study, the LDL-cholesterol level were similar among the Hp phenotype in both study cohorts, suggesting that the observed relationship between Hp phenotype and incident AMI was independent of LDL cholesterol levels.

Our finding is consistent with the work by Wang et al. who reported that Hp 1 allele is associated with macroangiopathy after adjusting for traditional risk factors [16]. Moreover, Wang et al. also observed correlation of Hp 1 allele with elevated level of serum haptoglobin, consistent with our previous report [23], and demonstrated higher level of oxidative stress marker, 8-hydroxy-2-deoxyguanosine (8-OHdG) in patients with Hp 1-1 genotype as compared to Hp 2-2 genotype. Furthermore, using Mendelian randomisation approach, they provided evidence of causal relationship between serum Hp and CVD. Our current work and study by Wang et al. were conducted in Chinese populations. Ethnic differences in the prevalence and risk of CVD, including AMI, among T2D patients are evident [46, 47]. The distribution of haptoglobin phenotype varies across ethnicity with Asians having lower frequency of Hp 1 allele compared to Europeans and Americans [11]. Importantly, these findings strengthen the role of Hp 1 allele as a risk factor for CVD in Asian population via unknown mechanism. It is tempting to postulate that the Hp phenotype may also interact with race/ethnicity in its association with CVD outcomes. Further trans-ethnic studies are needed to testify this hypothesis.

Series of studies have shown that Hp 1-1 genotype also confer risk of cerebrovascular disease in both type 1 [15, 48] and type 2 diabetes [49] and may be dependent on hypertension status at baseline. In our study, the prevalence of hypertension did not differ according to the Hp phenotype. Additionally, in a 10 year follow-up study, De Bacquer et al. showed that individuals with Hp 1-1 were double at risk of CHD mortality [50]. Understandably, the pathogenesis of CVD varies across arteries [51] and it is likely that Hp might play a different role in the proatherogenic progression.

The prospective design, large sample of well-characterised T2D patients and ascertainment of incident AMI via linkage to SMIR are the major strengths of the present study. However, certain limitations have to be acknowledged. This study was conducted in patients attending a regional hospital, thus may limit the generalizability of our findings. To overcome statistical limitations, this study combined findings from two separate cohorts. Several differences in baseline characteristic existed between participants from two cohorts. However, the clinical profiles among Hp groups within cohorts were mostly similar. Differences in relationship between HbA1c and haptoglobin phenotype in the two cohort and proportion of insulin users between two cohorts (SMAR2TD = 25.7% vs. DN = 31.9%) were observed and could be due to difference in the assays used for measuring HbA1c. Importantly, the association of Hp phenotype with incident AMI is independent of HbA1c levels and usage of insulin and difference in relationship between HbA1c and Hp phenotype across two cohorts may not confound the primary analysis. In this study, we only evaluated the association of Hp common phenotype. ELISA for Hp phenotyping compared to PCR gel-electrophoresis or TaqMan PCR assay is more sensitive and specific [22]. However, this approach (1) will not be able to detect rare alleles such as Hp-del allele and (2) has relatively lower sensitivity for Hp 1-1 (91.0%) compared to Hp 2-1 (97.2%) or Hp 2-2 (99.1%). Data from this study suggest presence of Hp 1 allele (Hp 1-1 or Hp 2-1) increases the incident AMI risk, notwithstanding the challenge of potential wrong assignment of Hp 1-1 individuals to Hp 2-1 phenotypes. Lastly, this is an observational study, which can support but not prove the causal relationship between Hp and AMI, and warrants future studies.

## Conclusions

Our study provides evidence that Hp 1-1 phenotype is a risk factor for incident AMI in type 2 diabetes patients in Chinese population, independent of traditional CVD risk factors. Further studies in independent cohort are warranted to validate our findings. Nevertheless, our findings demonstrate potential opportunities using Hp phenotype on patient stratification for effective management of CVD in type 2 diabetes population.

## Additional file

**Additional file 1: Table S1.** Baseline participant characteristic by study group. The units for Age and diabetes duration to be changed to (years)

## Data Availability

The datasets generated during and/or analyzed during the current study are available from the corresponding author on reasonable request.

## Abbreviations

Hp: haptoglobin
CVD: cardiovascular diseases
AMI: acute myocardial infarction
HR: hazard ratio
CI: confidence interval
8-OHdG: 8-hydroxy-2-deoxyguanosine
BMI: body mass index
TG: triglycerides
HDL: high-density lipoprotein
LDL: low-density lipoprotein
