# Supplementary material for: Association of haptoglobin phenotype with incident acute myocardial infarction in Chinese patients with type 2 diabetes
Source: Cardiovasc Diabetol. 2019 May 30;18:65. doi: 10.1186/s12933-019-0867-4 (PMC6542096; doi:10.1186/s12933-019-0867-4)
Supplement: Supplementary file 1 — Additional file 1: Table S1. Baseline participant characteristic by study group. The units for Age and diabetes duration to be changed to (years) [file 12933_2019_867_MOESM1_ESM.docx]

| **Variable** | **SMART2D** | **DN** | **P** |
| --- | --- | --- | --- |
| N | 1034 | 1290 |  |
| HP genotype (%) | - | - | 0.966 |
| HP 1-1 | 110 (10.6) | 133 (10.3) | - |
| HP 2-1 | 449 (43.4) | 561 (43.5) | - |
| HP 2-2 | 475 (45.9) | 596 (46.2) | - |
| Age (y) | 58.80 ± 11.43 | 58.43 ± 12.49 | 0.458 |
| Female (%) | 45.7 | 37.7 | **5.20E-05** |
| *Smoking History (%)* | - | - | **3.59E-19** |
| Current | 8.4 | 15.2 | - |
| Ex | 7.9 | 16.4 | - |
| Never | 83.7 | 70.6 | - |
| BMI (kg/m^2^) | 26.47 ± 4.68 | 26.14 ± 4.81 | 0.116 |
| HbA1c (%) | 7.76 ± 1.55 | 8.20 ± 1.88 | **2.39E-16** |
| Diabetes duration (y) | 10.0 (5.0-20.0) | 10.0 (5.0-18.0) | 0.822 |
| SBP (mm Hg) | 142.10 ± 19.37 | 135.85 ± 19.56 | **2.97E-14** |
| DBP (mm Hg) | 78.63 ± 9.44 | 76.74 ± 10.74 | **7.00E-06** |
| TC (mmol/L) | 4.40 ± 0.94 | 4.63 ± 1.12 | **1.89E-07** |
| HDL (mmol/L) | 1.33 ± 0.36 | 1.29 ± 0.37 | **0.003** |
| LDL (mmol/L) | 2.71 ± 0.81 | 2.74 ± 0.83 | 0.436 |
| TG (mmol/L) | 1.39 (1.02-1.96) | 1.49 (1.07-2.13) | **0.002** |
| uACR (mg/g) | 24.0 (7.0-117.8) | 38.3 (11.0-216.3) | **<0.001** |
| eGFR (ml/min/1.73m^2^) | 91.5 (68.2-103.5) | 83.8 (54.7-99.9) | **<0.001** |
| Medication (%) |  |  |  |
| Insulin | 265 (25.7) | 411 (31.9) | **0.001** |
| RAS antagonist | 620 (60.5) | 828 (64.2) | 0.068 |
| Lipid lowering | 831 (80.4) | 951 (74.1) | **2.92E-04** |

**Table S1.** Baseline participant characteristic by study group.

**Abbreviations**: BMI, body mass index; DBP, diastolic blood pressure; HDL-C, high-density lipoprotein cholesterol; LDL-C, low-density lipoprotein cholesterol; RAS, renin-angiotensin system; SBP, systolic blood pressure; TC, total cholesterol; TG, triglycerides; uACR, urine Albumin-to-Creatinine Ratio; eGFR, estimated glomerular filtration rate. Data are presented as frequencies (%) for categorical variables and as the mean + SD for continuous, normally distributed variables. P-values are for the difference between the cohorts.
